# Tongue coating microbiome data distinguish patients with pancreatic head cancer from healthy controls

**DOI:** 10.1080/20002297.2018.1563409

**Published:** 2019-01-28

**Authors:** Haifeng Lu, Zhigang Ren, Ang Li, Jinyou Li, Shaoyan Xu, Hua Zhang, Jianwen Jiang, Jiezuan Yang, Qixia Luo, Kai Zhou, Shusen Zheng, Lanjuan Li

**Affiliations:** a State Key Laboratory for Diagnosis and Treatment of Infectious Diseases, Collaborative Innovation Center for Diagnosis and Treatment of Infectious Diseases, The First Affiliated Hospital, School of Medicine, Zhejiang University, Hangzhou, Zhejiang, P.R. China; b Key Laboratory of Combined Multi-Organ Transplantation, Ministry of Public Health, Department of Hepatobiliary and Pancreatic Surgery, The First Affiliated Hospital, School of Medicine, Zhejiang University, Hangzhou, Zhejiang, P.R. China; c Department of Infectious Diseases; Precision Medicine Center, The First Affiliated Hospital of Zhengzhou University, Zhengzhou, Zhejiang, P.R.China; d Department of Geriatrics, The First Affiliated Hospital, College of Medicine, Zhejiang University, Hangzhou, Zhejiang, P.R. China; e Health Management Center, the First Affiliated Hospital, School of Medicine, Zhejiang University, Hangzhou, Zhejiang, China

**Keywords:** Pancreatic head carcinoma, microbiome dysbiosis, tongue coat, Miseq sequencing

## Abstract

**Background:** The microbiota plays a critical role in the process of human carcinogenesis. Pancreatic head carcinoma (PHC)-associated tongue coating microbiome dysbiosis has not yet been clearly defined.**Objective:** Our aim is to reveal the bacterial composition shifts in the microbiota of the tongue coat of PHC patients.**Design:** The tongue coating microbiota was analyzed in 30 PHC patients and 25 healthy controls using 16S rRNA gene sequencing technology.**Results:** The microbiome diversity of the tongue coat in PHC patients was significantly increased, as shown by the Shannon, Simpson, inverse Simpson, Obs and incidence-based coverage estimators. Principal component analysis revealed that PHC patients were colonized by remarkably different tongue coating microbiota than healthy controls and liver cancer patients. Linear discriminant analysis effect size revealed that *Leptotrichia*, *Fusobacterium*,*Rothia*, *Actinomyces*, *Corynebacterium*, *Atopobium*, *Peptostreptococcus*, *Catonella*, *Oribacterium*, *Filifactor*, *Campylobacter*, *Moraxella* and *Tannerella* were overrepresented in the tongue coating of PHC patients, and *Haemophilus*, *Porphyromonas* and *Paraprevotella* were enriched in the tongue coating microbiota of healthy controls. Strikingly, *Haemophilus*, *Porphyromonas*, *Leptotrichia* and *Fusobacterium* could distinguish PHC patients from healthy subjects, and *Streptococcus* and SR1 could distinguish PHC patients from liver cancer patients. **Conclusions:** These findings identified the microbiota dysbiosis of the tongue coat in PHC patients, and provide insight into the association between the human microbiome and pancreatic cancer.

## Introduction

Pancreatic carcinoma (PC) is the seventh highest cause of death from cancer worldwide. The symptoms of PC do not usually appear in the early stages of the disease [1] and therefore most patients already have advanced stage disease by the time they seek medical help. Many potential biomarkers in the blood and tumor tissue have been reported [2,3], but only early detection biomarkers have clinical value in terms of PC prevention and the identification of high-risk groups. For example, CA19-9 (carbohydrate antigen 19.9), which is used as a PC marker in clinic, lacks sensitivity and specificity, and is used for following known cases rather than for diagnosis [4]. The human microbiome has been shown to be closely associated with carcinogenesis [5] and tumor-promoting inflammation [6]. Seeking novel biomarkers from the human microbiome as early warning and diagnostic indicators has been a strategy employed for many diseases [7]. Therefore, research into the association between the microbiome and pancreatic cancer may open new opportunities to develop biomarkers to identify high-risk individuals.

Studies on the microbiome of PC patients have been focused on saliva [8], duodenal mucosa [9] and feces [10], and the results have shown dramatically different saliva, duodenal mucosa and fecal microbiota structure in PC patients compared with controls. However, the characteristics of the tongue coating microbiota of patients with PC have not yet been clearly defined. In the principal diagnostic methods of traditional Chinese medicine, tongue inspection can reveal the state of organ function and the progression of disease [11]. Our previous study showed that the tongue coating microbiome is unique among other human body microbiomes, and revealed two specific genera, *Oribacterium* and *Fusobacterium*, that could distinguish liver cancer (LC) patients from healthy subjects [12]. Many factors can result in variations in the tongue coating microbiota composition, for example, diet, race, oral hygiene, cancer site, and the environment. Therefore, we used 16S rDNA sequencing technology to demonstrate the tongue coating microbiota characteristics of patients with pancreatic head carcinoma (PHC, one of pancreatic adenocarcinoma which occurs in the head of the pancreas). Our findings would provide insight into the association between the human microbiome and PHC, and the development of new precautionary or diagnostic methods (i.e. eradication) for patients with PHC.

## Materials and methods

### Ethics statement

This study was approved with our previous project [13] by the Institutional Review Board of the First Affiliated Hospital, School of Medicine, Zhejiang University (reference number 2014-336), and the study was performed in accordance with the Helsinki Declaration and Rules of Good Clinical Practice. All participants approved and signed written informed consents upon enrollment.

### Subjects and sampling

The 16S rDNA sequence data of the tongue coat microbiome of patients with LC and healthy controls reported in our previous study [12] were used for comparison. We enrolled matched pancreatic head carcinoma (PHC) patients from the cohorts that participated in our previous project [13] to provide tongue coat samples. PHC was diagnosed by endoscopic ultrasonography and histopathologic examination at the hospital admission service center in the First Affiliated Hospital, College of Medicine of Zhejiang University, China. To avoid factors that may provoke an alteration in the tongue coat microbiome, such as medical intervention, the tongue coat samples were taken from each enrolled subject as close as possible to the time of enrolment. Tongue coat samples were collected and pretreated as previously reported [12]. Briefly, the coat samples were collected using a tongue scraper, immersed in phosphate-buffered saline, transferred to the laboratory, shaken, centrifuged immediately, and the supernatant was discarded. The tongue coat samples were discarded if (i) patients had a tumor beyond stage I (clinical Staging System on TNM Classification for PC); (ii) patients were aged <45 or >65 years, and had obstruction of the common bile duct; (iii) patients suffered from diseases of other organs, infectious diseases, or severe complications of other organs found by later examination; (iv) patients had periodontitis (with one of the symptoms in the tissues surrounding the teeth as follows: red, swollen, painful, bleeding gum, loose teeth and bad breath), cavities, canker sores and other oral diseases; and (v) patients who had been administered antibiotics or other drugs within the 3 months prior to enrolment. Finally, we recruited and included 30 PHC patients with tumors classified as stage I. In addition, the inclusion and exclusion enrollment criteria of 25 matched healthy controls and 35 patients with liver cancer were described in our previous study [12]. Written informed consent and questionnaires addressing previous and current diseases, lifestyles and medication (Supplementary table S1), were obtained from all subjects who voluntarily provided tongue coat samples. Each subject brushed his/her teeth in the morning and evening every day to maintain good oral hygiene. Each patient was given routine examinations (including oral examination) on the first day of admission, and the tongue coating was sampled at morning of the next day before brushing teeth and having breakfast. No one involved in the study had received antibiotics, probiotics or both in the eight weeks prior to enrollment, or had consumed/used any unhealthy substances (including alcohol, cigarettes/tobacco or drugs).

### DNA extraction, PCR and sequencing

The protocols for DNA extraction, V3–V4 amplification and sequencing were as described in our previous study [12]. Briefly, microbial DNA extracted using the Qiagen Mini Kit (Qiagen, Hilden, Germany), was quantified using a Qubit 2.0 Fluorometer (Invitrogen, Carlsbad, CA), and diluted to 10 ng/μL for PCR amplification in a thermocycler (Eppendorf Mastercycler). DNA libraries were constructed according to the manufacturer’s instructions, and DNA sequencing was performed on the Illumina MiSeq 2000 platform (Shallowater, USA) at the State Key Laboratory for Diagnosis and Treatment of Infectious Diseases (Zhejiang University, Hangzhou, China) according to standard protocols. The raw reads were deposited into the European Nucleotide Archive database (study accession no. PRJEB 27531).

### Sequence assembly and analysis

The 16S rDNA sequence data of the tongue coat microbiome of patients with LC and healthy controls reported in our previous study [12] were used for comparison. Sequence assembly and analysis were carried out as described previously [12]. Briefly, sequences were trimmed to 200 bp and merged into a single sequence using FLASH v1.2.10 software. Operational taxonomic units (OTUs) were classified based on 97% similarity after chimeric sequences had been removed using the UPARSE values (version 7.1 http://drive5.com/uparse/) [14]. The phylogenetic affiliation of each 16S rRNA gene sequence was analyzed by the RDP Classifier (http://rdp.cme.msu.edu/) [15] against the Silva (SSU123) 16S rRNA database using a confidence threshold of 70%.

Bacterial diversity was determined using a sampling-based analysis of OTUs and displayed as a rarefaction curve. Bacterial richness and diversity across the samples were calculated using the following indexes: Shannon, Simpson, inverse Simpson (invsimpson), Obs, Chao 1 and ACE [16]. To equalize the diﬀerence in sequencing depth of each sample, the sequences of all samples were downsized to 2,100 (20 permutations) [17]. A nonparametric Mann–Whitney U test was used to test for two groups, and the Kruskal–Wallis H test was used for three groups. Principal component analyses using weighted and unweighted UniFrac distance metrics were conducted. Principal coordinate analysis (PCoA) was also used to explain the variation in microbial communities between two groups based on weighted UniFrac values [18]. The R package (http://www.R-project.org/) was used to visualize the interactions among the bacterial communities of different samples [19].

The specific characterization of the fecal microbiota to distinguish taxonomic types was also analyzed via a linear discriminant analysis (LDA) effect size (LEfSe) method (http://huttenhower.sph.harvard.edu/lefse/) [20]. Applying a normalized relative abundance matrix, LEfSe was used to identify key bacteria in tongue coating samples of the patient group and in those of healthy controls at multiple levels in datasets, grade the key bacteria according to the results of a Mann–Whitney U-test, which determines the features with significantly different abundances between assigned taxa and uses LDA to assess the effect size of each feature [21], and visualize the results using taxonomic bar charts and cladograms. The *P* values were adjusted as described by Benjamini and Hochberg [22]. The Random forest [23] and Wilcoxon rank sum test were used to select differential species between PC patients and healthy controls, and between PC and LC patients with both the value of Mean_decrease_in_accuracy above 0.001, and *P* < 0.05 by the Wilcoxon rank sum test [17]; and to verify the key discriminatory OTUs which selected by random forest analysis, a 10-fold cross-validation analysis has been performed using rfcv function in R-package ‘randomForest’ (R version 3.2.1). Ten times cross-validation analysis was performed to sift through the minimum OTU combination with the lowest error rate and the lowest number that can accurately separate the two groups; and Receiver operating characteristics (ROC) analysis was then performed to measure the quality of the classification models by the R software package pROC [24].

## Results

### Clinical characteristics of the participants

After applying strict inclusion and exclusion criteria, we finally enrolled 30 patients with stage I PHC and 25 healthy subjects. The data for all subjects, including the clinical characteristics collected from medical records, are shown in Table 1. No significant differences existed between the groups in terms of age, gender, body mass index (BMI) and serum liver function. Clinically significant increases in the serum markers CEA, CA199 and CA125 were observed in the patients group.

**Table 1.** Clinical information in patients with pancreatic head carcinoma and healthy controls.

		Pancreatic head carcinoma		Healthy controls		
Clinical and pathological indexes		*N* = 30	%	*N* = 25	%	*P*
Age (year)		50.80 ± 5.33		48.16 ± 6.03		0.09*
Gender	Female	9	30.0	5	20.0	0.54^#^
	Male	21	70.0	20	80.0	
BMI (kg/m^2^)		22.51 ± 1.18		22.56 ± 1.63		0.90
Tumor size (cm)	≦2	13	43.3			
	2<&≦4	17	56.7			
ALT (5–40 U/L) mean ± SD		20.60 ± 17.44		19.36 ± 6.85		0.15
AST (8–40 U/L) mean ± SD		24.10 ± 12.76		20.60 ± 4.95		0.10
Albumin (35.0–55.0 g/L) mean ± SD		40.22 ± 4.04		49.03 ± 2.17		0.27
Total bilirubin (0–21 μmol/L) mean ± SD		16.63 ± 4.55		13.48 ± 4.89		0.27
Direct bilirubin (0–5 μmol/L) mean ± SD		4.29 ± 1.37		4.60 ± 1.73		0.39
CEA (0.0–5.0 ng/mL) Median(min-max)		2.31 (0.8–178.1)		1.87 (0.5–21)		<0.01
CA 199 (0.0–37.0 U/ml) Median(min-max)		50.4 (2–8130.4)		8.09 (3–47.3)		<0.01
CA 125 (0.0–35.0 U/ml) Median(min-max)		34.1 (4.3–96.6)		6.8 (2.5–31.5)		<0.01

The continuous variables were presented as mean ± SD; *Independent t-test; ^#^Pearson Chi-Square test or Fisher exact test. Abbreviations: BMI: body mass index; ALT: alanine aminotransferase; AST: aspartate aminotransferase; CEA: carcinoembryonic antigen; CA 199: carbohydrate antigen 19–9; CA 125: carbohydrate antigen 12–5.

### Differences in the tongue coating microbiota between PCH patients and healthy subjects

**Figure 1.**
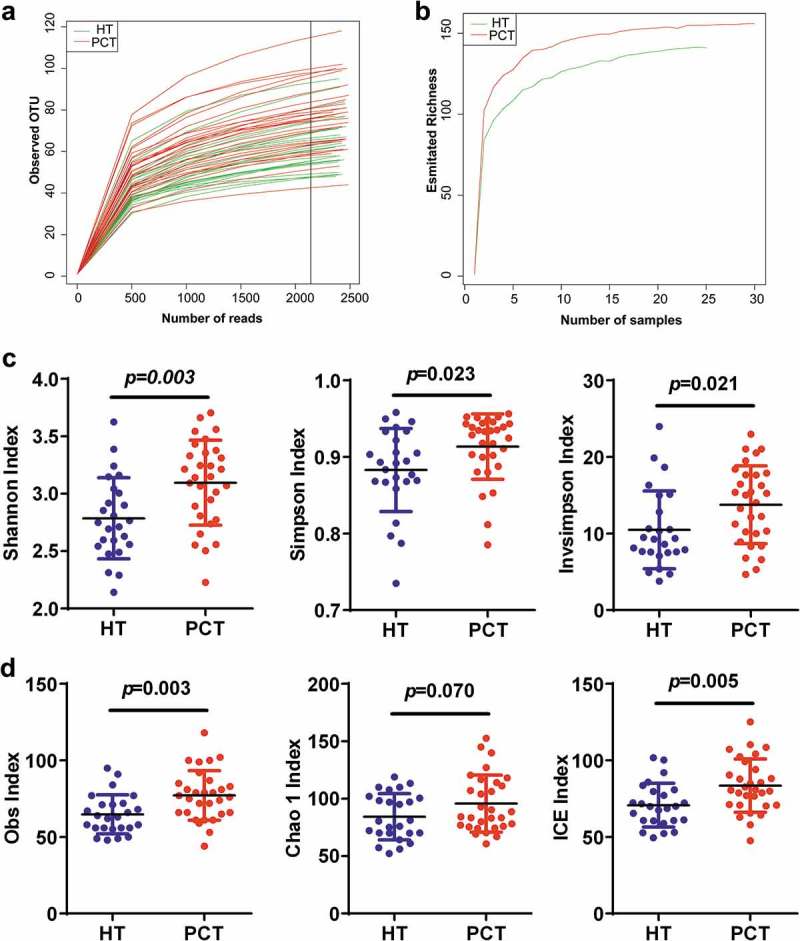
Phylogenetic diversity of tongue coating microbiota among individuals and between PC patients and healthy subjects. (a) Rarefaction analysis of bacterial 16S rRNA gene sequences was used to evaluate if further sequencing would likely detect additional taxa, indicated by a plateau. (b) Richness index curves that evaluate the number of samples likely required to identify additional taxa indicated by a plateau. (c) Scatterplots depict microbiota diversity differences according to the Shannon index, Simpson index and invsimpson index between the PCT and HT. (d) Scatterplots depict microbiomes diversity differences according to the Obs index, Chao 1 index and ICE index between PCT and HT. Plot parameters, the ‘black line’ symbol represents median value, and the upper and lower ranges of the plots represent the 75% and 25% quartiles, respectively. PCT, pancreatic cancer patients tongue coat; HT, healthy subjects tongue coat.

After merging and filtering, 261,280 sequence reads were generated. A total of 207 bacterial OTUs were identified across the entire cohort. In total, 0.976 and 0.916 of all reads could be assigned to the family and genus level, respectively (Supplementary datasets S1_a). According to the rarefaction data (Figure 1(a)), subsets of 2,100 reads (the number was sufficient to identify most of the bacterial community members of each tongue coat microbiome because it is the point where the rarefaction curve of the observed OTUs reached a plateau) were picked randomly to normalize sequencing depth for subsequent community composition analysis. For each sample, the rarefaction curves of the richness index curves for each of the two cohorts plateaued (Figure 1(b)), indicating that almost all of the OTUs present in each group had been detected. A total of 158 bacterial OTUs were identified across the entire cohort, and classified into 12 distinct bacterial phyla, 52 distinct bacterial families and 103 distinct bacterial genera (Supplementary datasets S1b). The species richness and diversity estimators were obtained for each microbiome (Supplementary datasets S1_c). With the exception of the Chao 1 index, the other community diversity and richness indexes (such as Shannon, Simpson, invSimpson, Obs and incidence-based coverage estimators) showed significant increases in the PHC tongue coating microbiome when compared with that of the healthy control microbiome. The Chao 1 index, as one of the community richness estimators, showed a slight increase but this was not statistically significant (*P* < 0.05) (Figure 1(c,d)).

**Figure 2.**
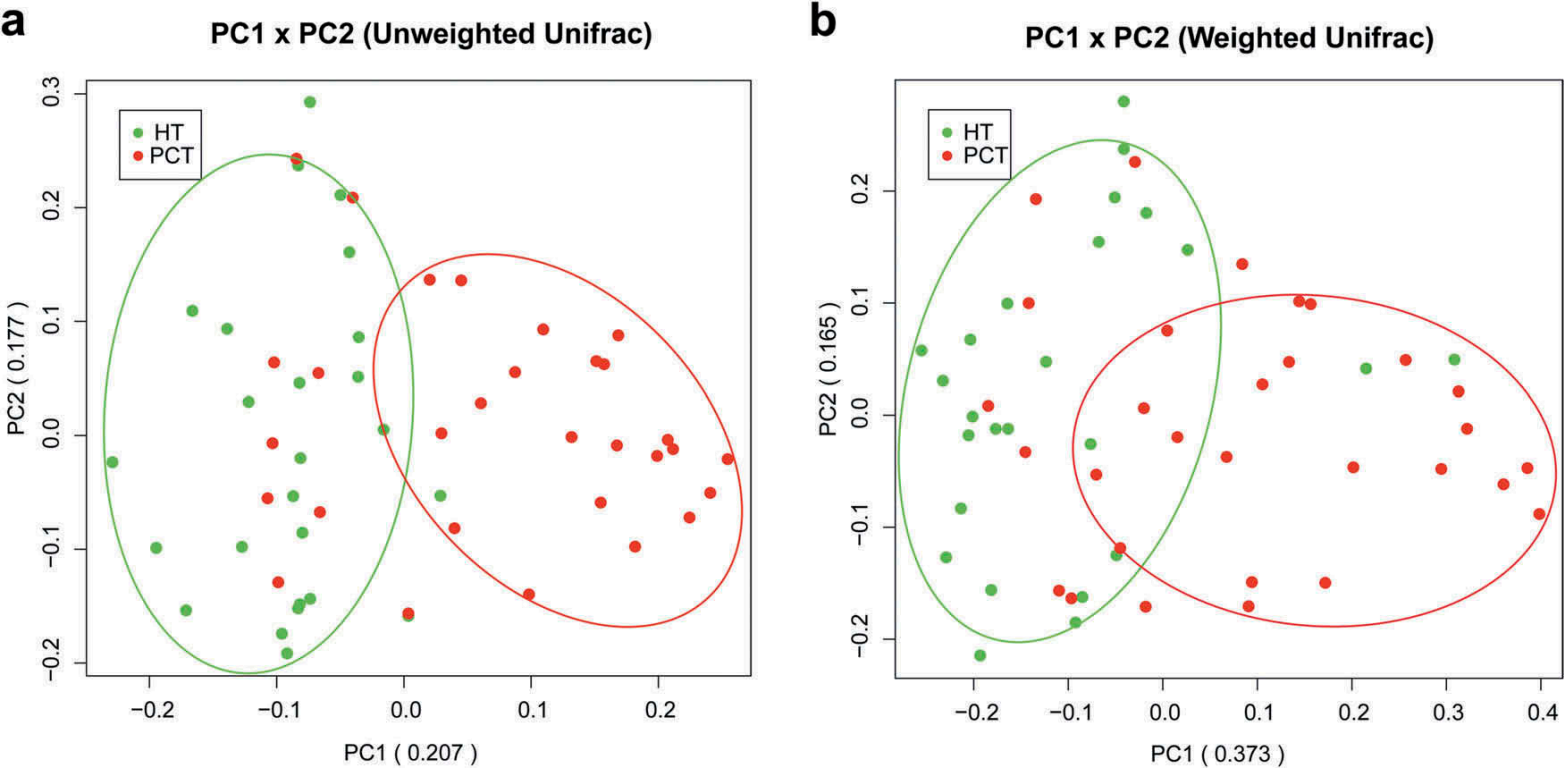
Bacterial diversity clustering by combining unweighted and weighted UniFrac PCoA of tongue coating microbiota. (a) Unweighted UniFrac (qualitative); (b) weighted UniFrac (qualitative). Each symbol represents a sample (blue, PCT; green, HT); the variance explained by the PCs is indicated in parentheses on the axes. PCT, pancreatic cancer patients tongue coat; HT, healthy subjects tongue coat.

**Figure 3.**
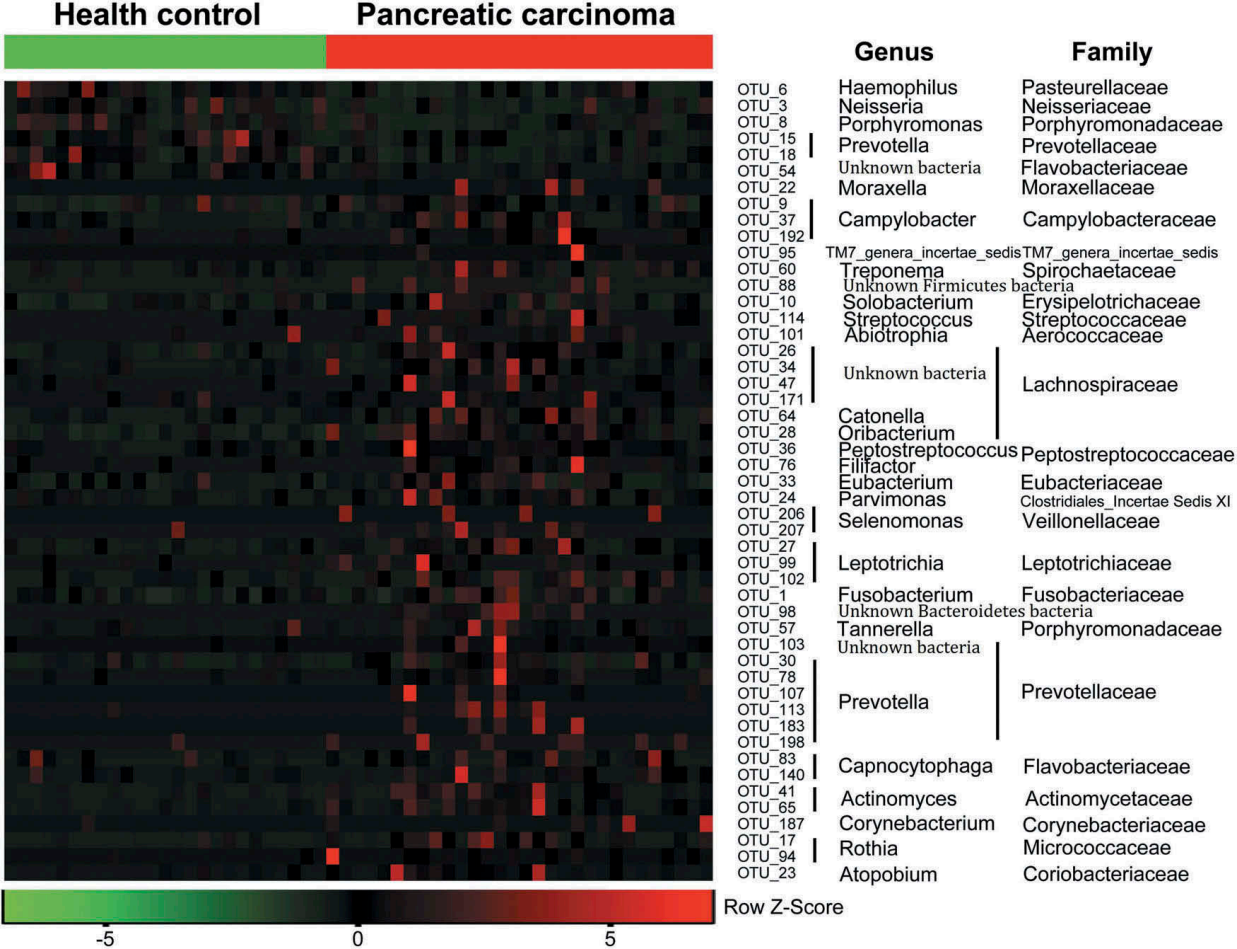
Heat maps of the relative abundances of the discriminatory OTUs that drive the differences between PCT and HT. For each sample, the columns show the relative abundance data of the discriminatory OTUs listed to the right of the figure. The relative abundance of each OTU was used to plot the heat map (blue, low abundance; red, high abundance). The group information was shown above the plot: healthy controls on the left with green line, PC patients on the right with red line. Each row represents one OTU. The family and genus of each key OTU are noted to the right of the figure. The top six OTUs were found enriched in healthy controls, and the rest OTUs enriched in pancreatic cancer patients. PCT, pancreatic cancer patients tongue coat; HT, healthy subjects tongue coat.

Both unweighted (Figure 2(a)) and weighted (Figure 2(b)) Unifrac principal component analyses revealed that PHC patients were colonized by significantly different tongue coating microbiota compared with healthy controls (*P* < 0.05 using PERMANOVA (R-vegan function adonis)). Similar results were obtained with PCoA using the Hellinger distance and Jensen–Shannon divergence analysis methods (Figure S1). Heatmap results delineated 49 distinguishing OTUs (assigned to 25 different genera and 24 different families) in the tongue coating microbiota of PHC patients and controls. Of these discriminatory OTUs, six were decreased, while 43 were increased in the PHC tongue coating microbiome, when compared with the tongue coating microbiome of healthy controls (Figure 3).

**Figure 4.**
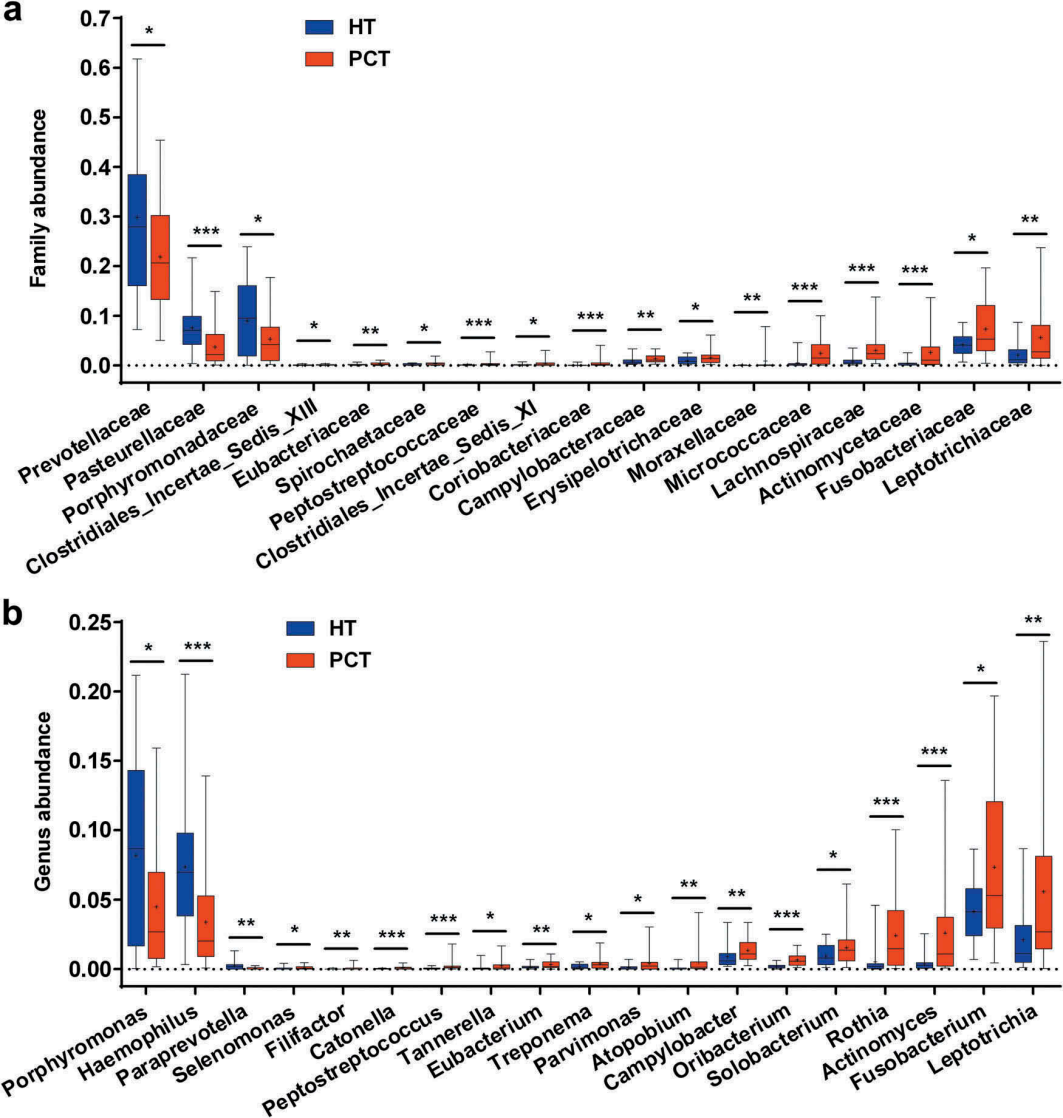
Comparison of the microbiome at the family (a) and genus (b) levels between PCT (red) and HT (green), respectively. Box parameters, the ‘+’ symbol represents median value, and the upper and lower ranges of the box represent the 75% and 25% quartiles, respectively; *P* values were calculated using the nonparametric Mann–Whitney test and were shown in Supplementary Datasets S2_b (Family) and_c (Genus); significant correlations by *P < 0.05; **P < 0.01 and ***P < 0.001. PCT, pancreatic cancer patients tongue coat; HT, healthy subjects tongue coat.

The phylum structure of the tongue coating microbiota for each participant is shown in Figure S2a. Of the major phyla, Bacteroidetes, Proteobacteria, Firmicutes, Fusobacteria, Actinobacteria and TM7 were the six most predominant, together accounting for more than 96% of the total sequences (Figure S2b). Analysis at the phylum level showed that PCH patient groups presented significantly higher relative abundance of Firmicutes, Fusobacteria and Actinobacteria (*P* < 0.05, *P* < 0.001 and *P* < 0.001, respectively, by the Mann–Whitney U-test), and a significantly lower relative abundance of Bacteroidetes (*P* < 0.001) when compared with the healthy control group (Figure S2c). At the family level, *Prevotellaceae, Pasteurellaceae* and *Porphyromonadaceae* were more abundant in the healthy control tongue coating microbiome, and 14 bacterial families were more abundant in the PHC tongue coating microbiome, including *Leptotrichiaceae, Fusobacteriaceae, Actinomycetaceae, Lachnospiraceae, Micrococcaceae, Erysipelotrichaceae* and *Campylobacteraceae* (Figure 4(a)). Of the 19 discriminatory genera, the relative abundance of *Porphyromonas, Haemophilus* and *Paraprevotella* were significantly higher in the healthy control tongue coating microbiome, and the others were significantly higher in the PHC tongue coating microbiome, including *Leptotrichia, Fusobacterium, Actinomyces, Rothia, Solobacterium, Oribacterium, Campylobacter, Atopobium* and *Parvimonas* (Figure 4(b)).

**Figure 5.**
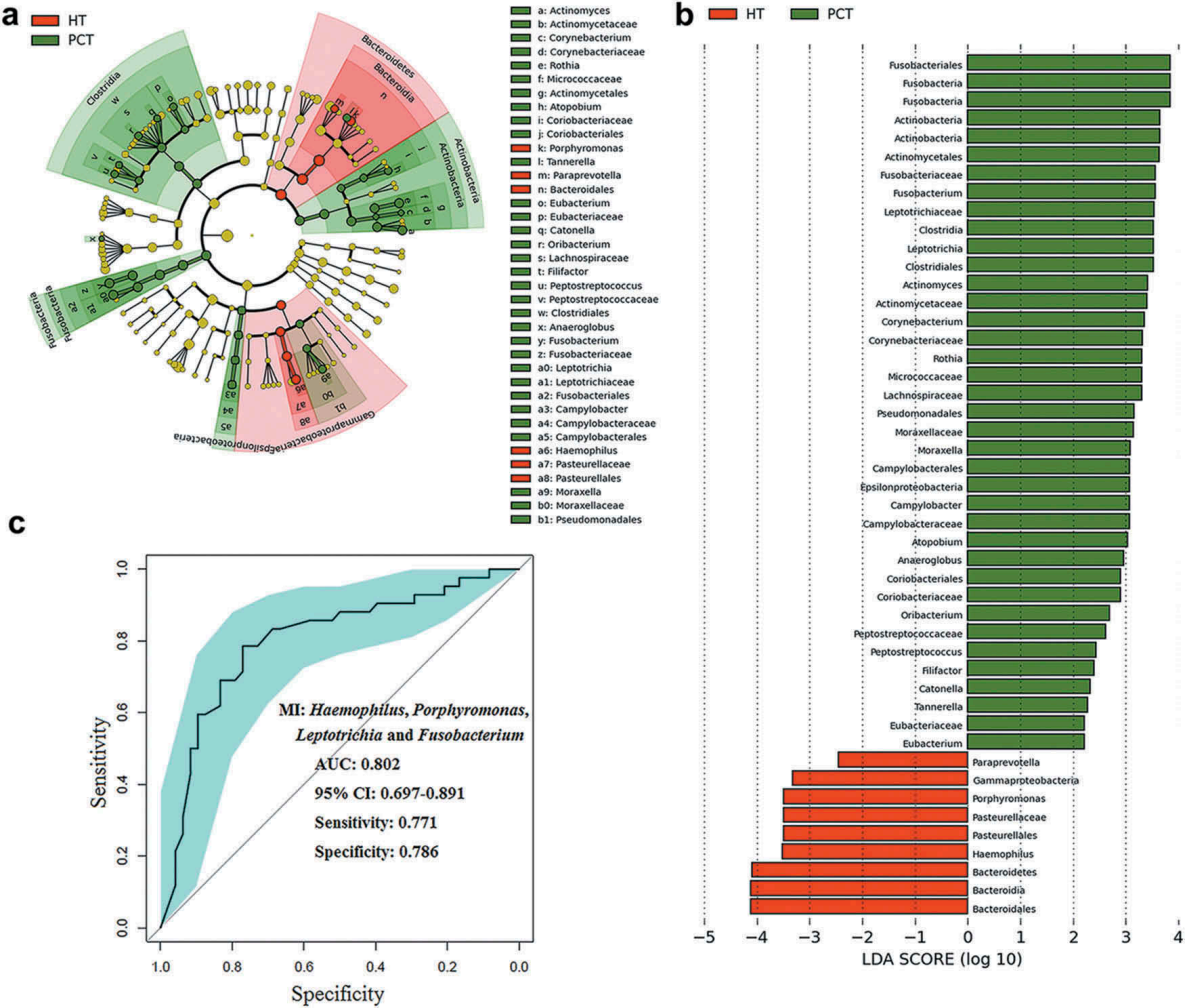
LEfSe and LDA analysis based on OTUs characterize microbiota between the PCT and HT. (a) Cladogram using the LEfSe method indicating the phylogenetic distribution of tongue coat microbes associated with patients with PC (green indicates phylotypes statistically overrepresented in PCT) and healthy subjects (red indicates phylotypes overrepresented in healthy subjects). Each filled circle represents one phylotype, and phylum and class are indicated in their names on the cladogram and the order, family, or genera are given on the right panel. (b) A histogram of the linear discriminant analysis (LDA) scores was calculated for the selected taxa which showed the significant bacterial difference between the PCT and HT. LDA score at the log_10_ scale is indicated at the bottom. The greater the LDA score is, the more significant the microbial biomarker is in the comparison. (c) Prediction of microbial index (MI, the key genera which can distinguish PC patients from healthy controls). The area under the ROC curve (AUC = 0.862) and microbial index (*Porphyromonas, Leptotrichia* and *Fusobacterium*) were calculated and is shown in the center. PCT, pancreatic cancer patients tongue coat; HT, healthy subjects tongue coat; MI, microbial index.

We also performed LEfSe to identify the discriminatory taxa in the tongue coating microbiota of PHC patients and controls based on the Ribosomal Database Project (RDP; http://rdp.cme.msu.edu/) taxonomy data (Figure 5(a,b)). The PHC patient tongue coating microbiome was characterized by a preponderance of *Fusobacterium, Leptotrichia, Actinomyces, Corynebacterium, Rothia, Moraxella* and *Atopobium* (LDA score (log10) > 3), whereas the healthy control tongue coating microbiome was characterized by a preponderance of *Porphyromonas* and *Haemophilus* (LDA score [log10] > 3). *Fusobacterium, Leptotrichia* and *Porphyromonas*, as microbial indexes, contributed to the differentiation between PCH and the healthy control tongue coating microbiota with an ROC (receiver operating characteristic curve)-plot AUC (the area under the parasitemia curve) value of 0.802, and values of 0.771 for sensitivity and 0.786 for specificity (Figure 5(c)).

### Differences in the tongue coating microbiota between PCH and LC patients

**Figure 6.**
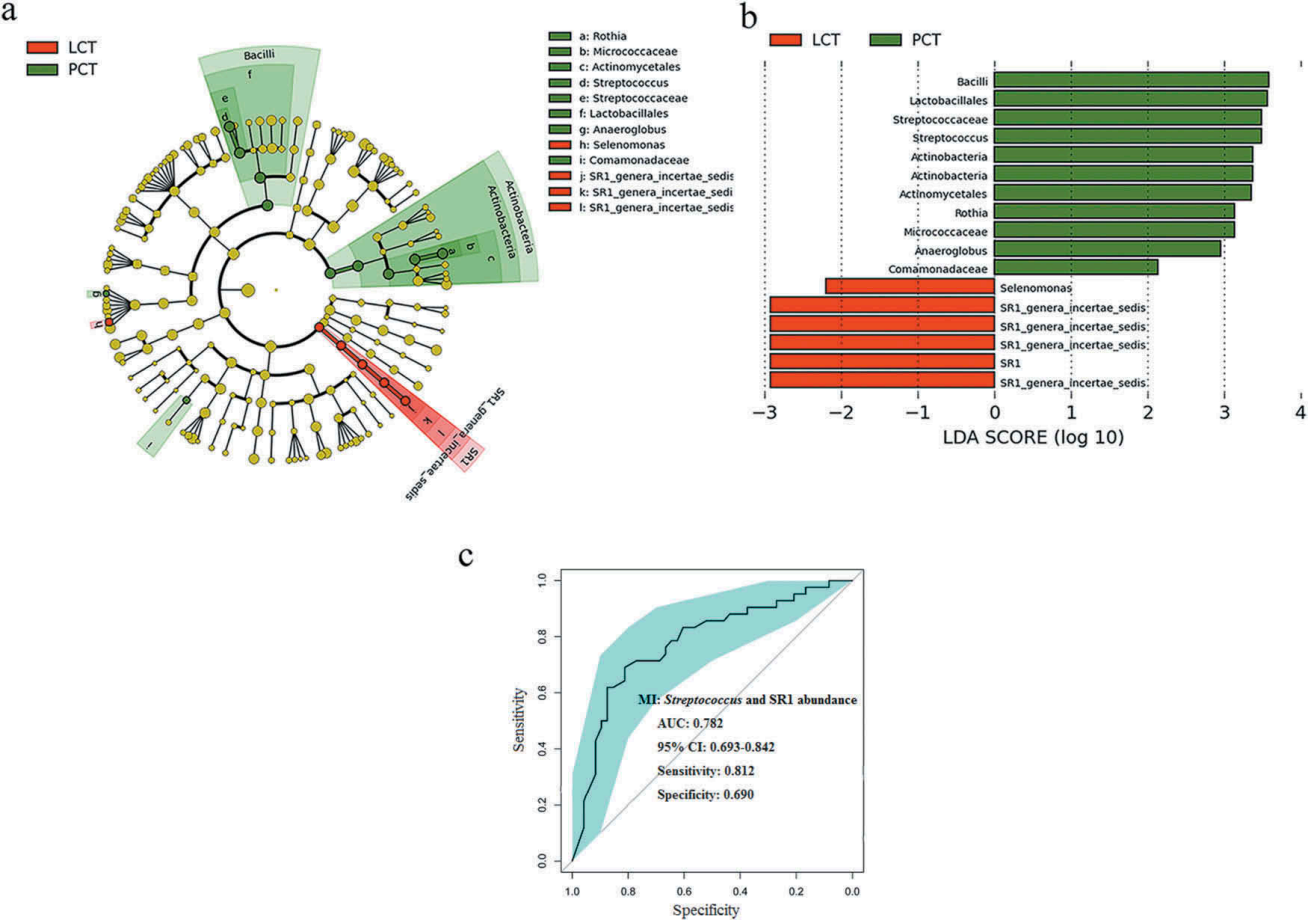
LEfSe and LDA analysis based on OTUs characterize microbiota between the PCT and LCT. (a) Cladogram using the LEfSe method indicating the phylogenetic distribution of tongue coat microbes associated with patients with PC (green indicates phylotypes statistically overrepresented in PCT) and LC (red indicates phylotypes overrepresented in LCT). Each filled circle represents one phylotype, and phylum and class are indicated in their names on the cladogram and the order, family, or genera are given on the right panel. (b) A histogram of the linear discriminant analysis (LDA) scores was calculated for the selected taxa which showed the significant bacterial difference between the PCT and LCT. LDA score at the log_10_ scale is indicated at the bottom. The greater the LDA score is, the more significant the microbial biomarker is in the comparison. (c) Significant differences in the abundance of predominant genera between the cancer patients and the healthy controls. The average abundance values for each bacterium is depicted as mean ±SEM. *P* values were calculated using the nonparametric Mann–Whitney test, and are shown in Supplementary table S2. Significant differences by *P < 0.05; **P < 0.01 and ***P < 0.001. HT, healthy subjects tongue coat; PCT, pancreatic cancer patients tongue coat; LCT, liver cancer patients tongue coat.

We used LEfSe to compare the estimated phylotypes of the tongue coat microbiota between PHC and LC patients (which had been deposited into the European Nucleotide Archive database under study accession number: PRJEB12503, and second study accession number: ERP013989). The PCoA results showed that the structure of the tongue coating microbiota in PHC patients was also different from that of LC patients (Figure S3). The relative abundance of *Streptococcus* and *Rothia* were higher in the tongue coating microbiota of PCH patients, while the relative abundance of SR1 was higher in that of LC patients (Figure 6(a,b)). The results of statistical analysis of these four discriminatory genera in the tongue coat microbiomes of PC and LC patients using the nonparametric Mann–Whitney U test at the genus level are shown in Supplementary table S2. *Streptococcus* and SR1, as microbial indexes, contributed to the differentiation between PCH and LC tongue coating microbiota with an ROC (receiver operating characteristic curve)-plot AUC (the area under the parasitemia curve) value of 0.782, and values of 0.812 for sensitivity and 0.690 for specificity (Figure 6(c)).

## Discussion

PC is an aggressive lethal cancer. Primary prevention of PC is of particular importance in reducing the burden of this malignancy. Consequently, it is critical to identify markers that can identify high-risk individuals. The microbiota plays a critical role in the process of carcinogenesis [25]. Therefore, research into the association between the microbiome and PC may provide new insight that may lead to the development of biomarkers to identify high-risk individuals. This study revealed previously undescribed bacterial composition shifts in the microbiota of the tongue coat of PHC patients, and the unique tongue coating microbial profiles may indicate potential microbial markers for noninvasive PHC. Because specific bacteria are associated with immunity, we propose that such microbiota dysbiosis may provide substrates that can stimulate or influence inflammatory processes in the pancreas.

To the best of our knowledge, this is the first study characterizing the PHC tongue coat microbiota both in composition and diversity. The relative abundance of Fusobacteria (*Leptotrichia* and *Fusobacterium*) was higher in PHC patients with the highest LDA score, followed by the phyla Actinobacteria (*Rothia, Actinomyces, Corynebacterium* and *Atopobium*), Clostridia (*Peptostreptococcus, Catonella, Oribacterium* and *Filifactor*) and Epsilonproteobacteria (*Campylobacter*), including two genera *Moraxella* in Gammaproteobacteria and *Tannerella* in Bacteroidetes. Members of these genera that were enriched in the PHC tongue coat microbiota were highly heterogeneous, and some of them have been recognized as opportunistic pathogens found to be associated with periodontitis and other diseases, such as *Leptotrichia* and *Atopobium* implicated in bacterial vaginosis [26], *Fusobacterium* in the development of colorectal cancer, the prognosis of pancreatic cancer [27] and also periodontitis [28], *Campylobacter* and *Rothia* in malignant oral leukoplakia [29], *Actinomyces* in chronic periodontitis [30], *Corynebacterium* in cardiac or pulmonary disease [31], *Filifactor* in periodontitis [32], *Campylobacter, Peptostreptococcus* and *Catonella* in chronic obstructive pulmonary disease and also periodontitis [33], *Oribacterium* in liver cancer [12], *Tannerella* in esophageal adenocarcinoma and also periodontitis [34], *Moraxella* in asthma [35] and also in the unspecific pro-immune response [36]. Furthermore, potentially pathogenic oral bacteria have attracted considerable attention as a risk factor for PC [37]. Changes in the saliva microbiota typical of periodontitis seem to be associated with an increased risk of developing PC. Among these discriminatory genera, *Fusobacterium, Actinomyces, Campylobacter, Atopobium, Oribacterium* and *Peptostreptococcus* were also found to be enriched in the tongue coat microbiota of LC patients (in total, 23 LC-discriminatory genera). A high abundance of many of these genera was associated with the cancerous microenvironment and disturbance of the host immune response, and was thought to play a role in the initiation and progression of cancer [38–42]. It was proposed that the inhibition of pathogenic bacterial growth via reestablishing the oral microecological balance might reduce the risk of PC. Additionally, we found that three genera associated with opportunistic pathogens such as *Haemophilus* (Gammaproteobacteria), *Porphyromonas* and *Paraprevotella* (both from the phyla Bacteroidetes) were less abundant in PHC patients than in the healthy controls. The relative decrease in the abundance of *Haemophilus, Porphyromonas* and *Paraprevotella* in the PHC tongue coating microbiota was accompanied by an increase in the population of *Moraxella* (Gammaproteobacteria) and *Tannerella* (Bacteroidetes), which ultimately led to a decrease in the population of the phyla Gammaproteobacteria and Bacteroidetes in the PHC tongue coat microbiota.

In the oral environment, dynamic microbial composition shifts in the tongue coat were found to be different from those in the saliva, as reported in previous studies. Firmicutes, Bacteroidetes, Proteobacteria, Actinobacteria and Fusobacteria, which were reported to be the major phyla present in the saliva microbiota of PC [8] were also identified as the top phyla in the tongue coat microbiota of PHC, but with the following phyla in descending order of abundance: Bacteroidetes, Proteobacteria, Firmicutes, Fusobacteria and Actinobacteria. In the saliva microbiota profiles of PC patients, the relative abundance of Proteobacteria was lower, whereas in the PHC tongue coat microbiota profiles, Bacteroidetes was lower than Proteobacteria, and Proteobacteria showed no difference, when compared with healthy control cohorts. The relative abundance of the other three top phyla in PHC tongue coat microbiota showed a significant increase, whereas in the saliva microbiota, they showed no significant difference when compared with healthy controls. In addition, *Neisseria elongata* and *Streptococcus mitis* in saliva were identified as biomarkers to distinguish PC cases from healthy controls with high sensitivity and specificity [43]. The relative abundance of both genera showed little difference in the tongue coating microbiome between patients and controls. The phenomenon reported by previous studies [44] that PC patients had significantly higher levels of the *Bacteroides* genus in their saliva compared with control subjects was also not replicated in the tongue coating microbiome; whereas the phenomenon of higher relative abundance of *Proteobacteria* in healthy individuals was replicated in our tongue coating microbiome study. Possible explanations for this inconsistency are the differences in tumor sites and the differences in locations of the microbiota. It has been reported that differences existed in the intestinal bacterial composition shifts between patients with PCH and those with pancreatic tail/body cancer [10].

It has been reported that the oral microbiota overlaps with the digestive tract microbiota [45] and can translocate to the pancreas through the circulation, and under disease conditions, such as liver cirrhosis, an association between dysbiosis in the gastrointestinal and pancreatic microbiota has been reported [46]. Using NGS technology, multiple studies have evaluated the saliva, fecal and cancer tissue microbiota of PC patients and the duodenal bacterial microbiota of patients with PHC. Shifts in composition of the tongue coat microbiota were much more complex in those of other human body microbiotas. Some genera that were enriched in the tongue coat microbiota were also found to be present in higher abundance in other body microbiotas of PC patients, with the exception of the duodenal microbiota. For example, *Campylobacter* and *Leptotrichia* were also found to be enriched in the fecal microbiota of PC patients [10], whereas only *Leptotrichia* was enriched in the saliva microbiota of PC patients [8], and *Fusobacterium* was detected in tumor tissues [47]. None of these discriminatory genera that were enriched in the tongue coat microbiota of PC patients were found to be enriched in the duodenal mucosa of PC patients. However, the duodenal mucosa, tongue coating and saliva microbiomes consistently showed a decrease in *Porphyromonas* compared with healthy controls [9]. It has been reported that a high level of antibodies to *Porphyromonas gingivalis* in the serum correlates with a lower risk of PC [37]. Perhaps, the phenomenon of *Porphyromonas* in the human microbiota with higher relative abundance aids anticancer-associated immunity.

Microbiota dysbiosis has been linked to immune disorders and has been associated with the inflammatory response that contributes to the development of cancer [25]. Conversely, cancer can result in fluctuations in the composition of the microbiota via a metabolic and immune imbalance. Higher levels of antibodies to oral bacteria were verified to be linked to a lower risk of PC [37]. Many studies have been published providing evidence that the oral or gut microbial index can distinguish PC patients from healthy cohorts [8,13]. In the present study, the PHC- discriminatory genera, *Porphyromonas, Haemophilus, Fusobacterium* and *Leptotrichia*, were shown to distinguish PC patients from the healthy cohorts. *Porphyromonas* and *Leptotrichia* in the saliva microbiome were identified as microbial biomarkers for PC [8]. *Fusobacterium* was also confirmed to be abundant in PC tumor tissue [47], whereas *Haemophilus* was found at lower levels in the tongue coating microbiota of LC patients. Moreover, the results derived from comparison between the PHC and LC tongue coat microbiotas using LEfSe and LDA analysis verified the specific link between tongue coat microbiota alterations and PHC.

## Conclusions

Complete characterization of the gastrointestinal, saliva and tongue coating microbiomes, and alterations in the microbiotic communities in PHC patients compared with healthy controls could potentially lead to the development of early diagnostic or preventive tools for PHC. Our results confirmed the association between microbial dysbiosis and pancreatic diseases. Low levels of *Haemophilus* and *Porphyromonas* and high levels of *Leptotrichia* and *Fusobacterium* in the tongue coat microbiota were the most striking differences observed between the tongue coat microbial profiles of PHC patients and healthy controls. The present study also revealed the different tongue coating bacterial compositions between PHC and LC patients. The variations in microbial composition and proinflammatory disorders are most likely keys to this association. However, further investigation is still required to confirm these findings in a large cohort in independent centers and to prove the causal association between the discriminatory bacteria and the risk of PHC.
