# Supplementary figures and images for: Tongue coating microbiome data distinguish patients with pancreatic head cancer from healthy controls
Source: J Oral Microbiol. 2019 Jan 28;11(1):1563409. doi: 10.1080/20002297.2018.1563409 (PMC6352935; doi:10.1080/20002297.2018.1563409)

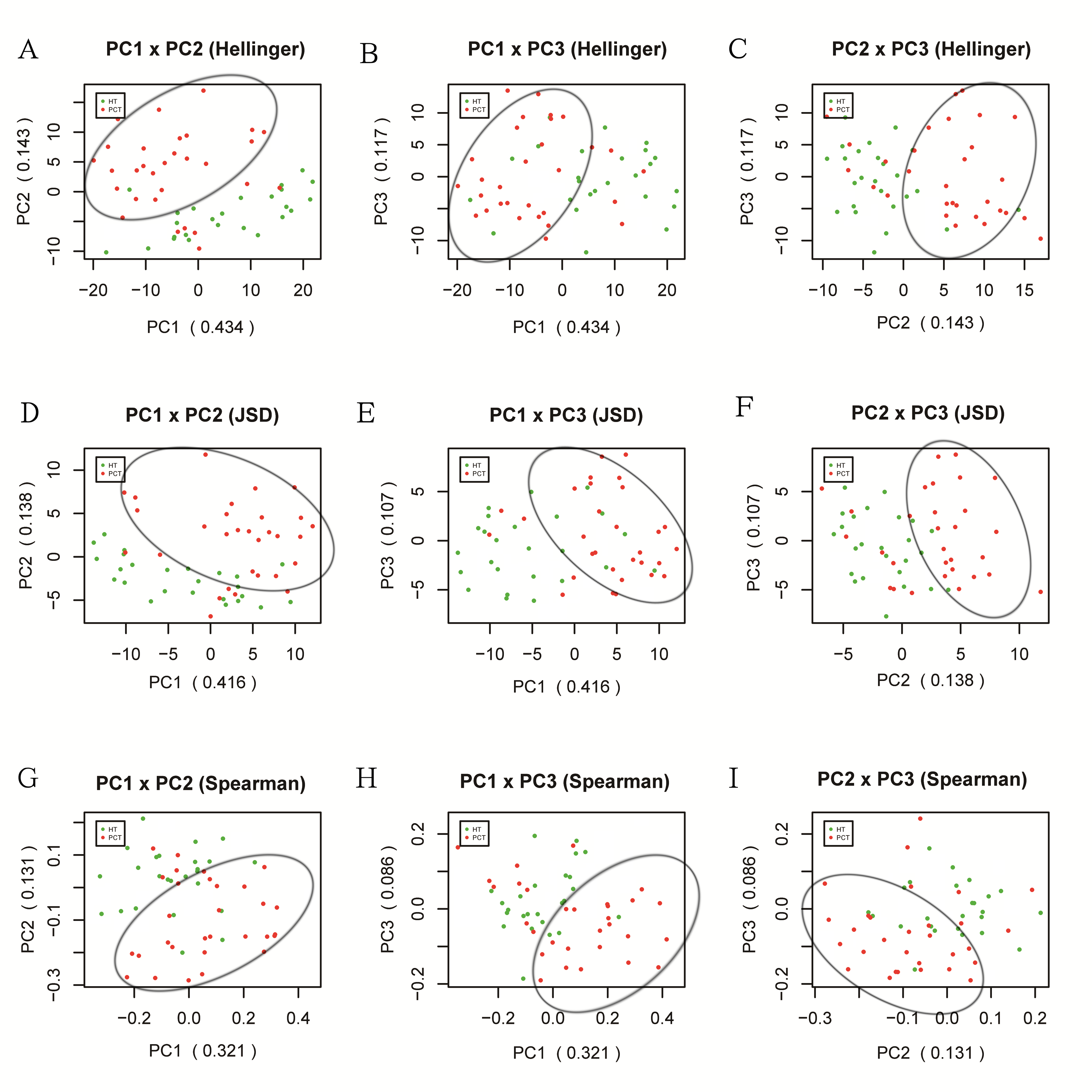

Supplement: Supplemental Material [file ZJOM_A_1563409_SM2802.zip › supp_Mat/Figure S1.tif]

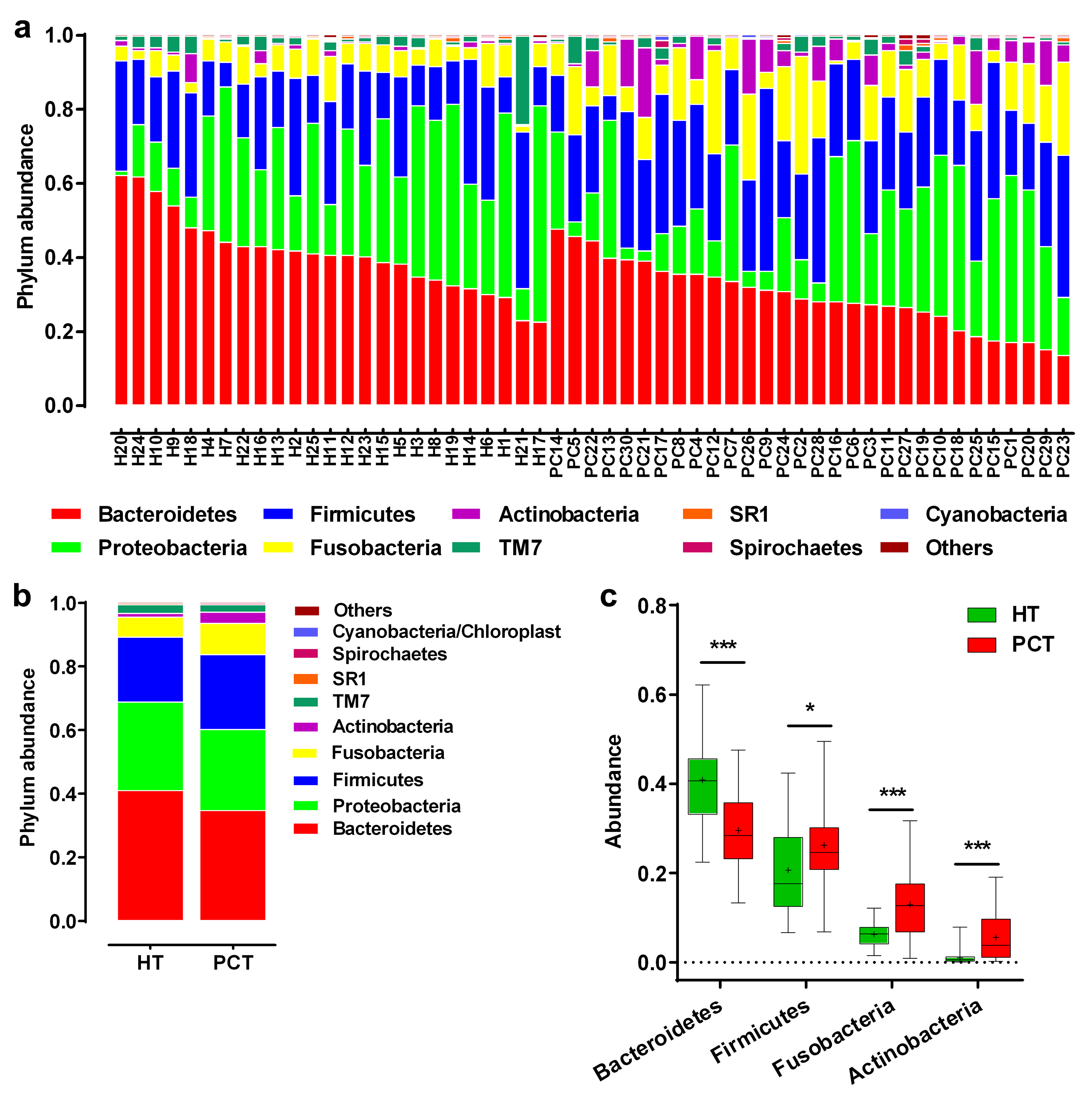

Supplement: Supplemental Material [file ZJOM_A_1563409_SM2802.zip › supp_Mat/Figure S2 bacterial composition.tif]

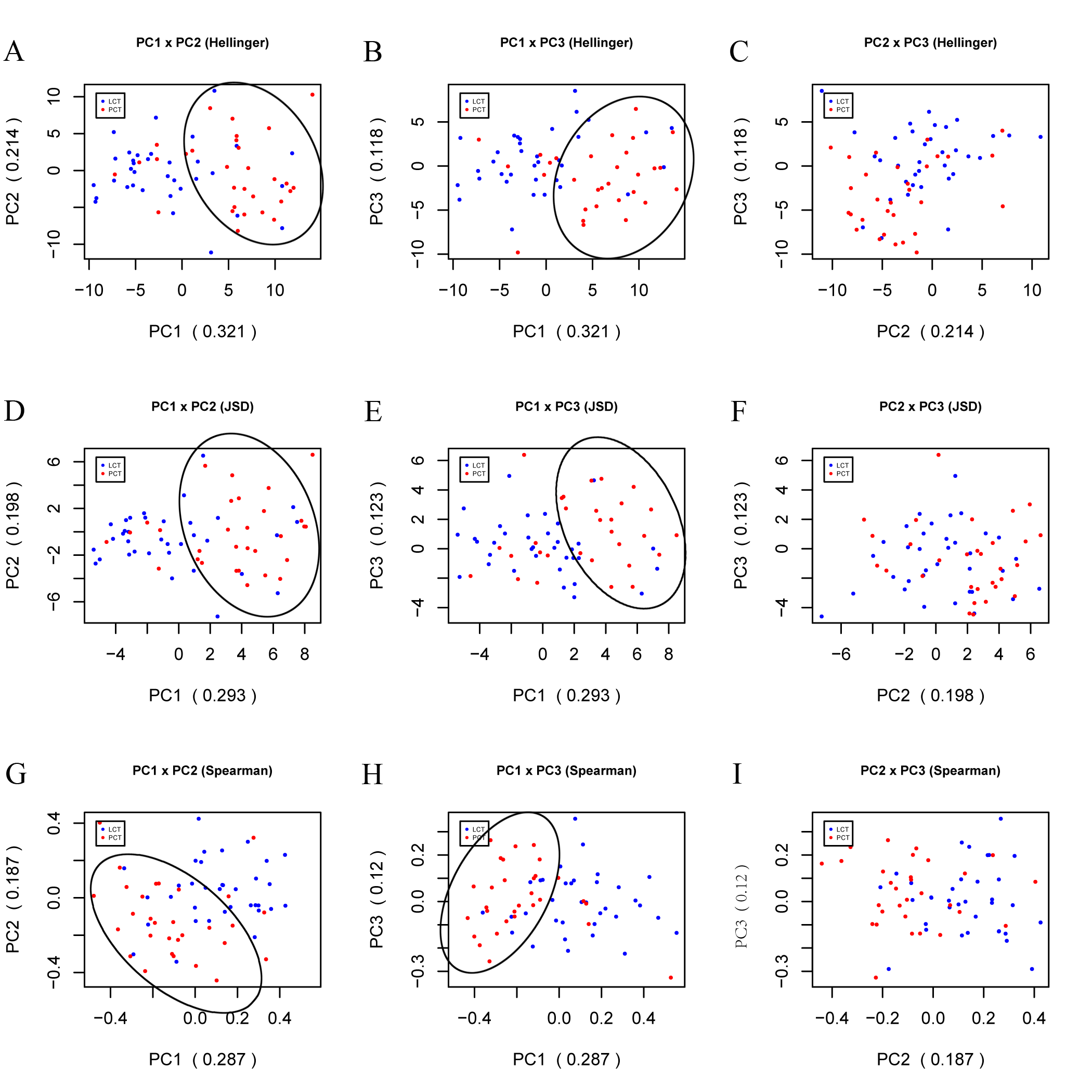

Supplement: Supplemental Material [file ZJOM_A_1563409_SM2802.zip › supp_Mat/Figure S3.tif]
