# Supplementary material for: Tongue coating microbiome data distinguish patients with pancreatic head cancer from healthy controls
Source: J Oral Microbiol. 2019 Jan 28;11(1):1563409. doi: 10.1080/20002297.2018.1563409 (PMC6352935; doi:10.1080/20002297.2018.1563409)
Supplement: Supplemental Material [file ZJOM_A_1563409_SM2802.zip › supp_Mat/Supplementary Information.doc]

Tongue coating microbiome data distinguish patients with pancreatic head cancer from healthy controls

Haifeng Lu ^1ǂ^, ZhigangRen^2,3ǂ^, Ang Li^2,3^, Jinyou Li^4^, Shaoyan Xu^2^, Hua Zhang^1^, Jianwen Jiang^2,5^, Qixia Luo^1^, Kai Zhou^1^, Shusen Zheng^2*^ & Lanjuan Li^1*^

^1^ State Key Laboratory for Diagnosis and Treatment of Infectious Diseases, Collaborative Innovation Center for Diagnosis and Treatment of Infectious Diseases, The First Affiliated Hospital, School of Medicine, Zhejiang University, Hangzhou 310003, P.R. China.

^2^ Key Laboratory of Combined Multi-organ Transplantation, Ministry of Public Health; Department of Hepatobiliary and Pancreatic Surgery, First Affiliated Hospital, School of Medicine, Zhejiang University, Hangzhou 310003, P.R. China.

^3^ Department of Infectious Diseases; Precision Medicine Center, the First Affiliated Hospital of Zhengzhou University, Zhengzhou 450052, P.R.China.

^4^ Department of Geriatrics, the First Affiliated Hospital, College of Medicine, Zhejiang University, Hangzhou310003, P.R. China.

^5^ Health Management Center, the First Affiliated Hospital, School of Medicine, Zhejiang University, Hangzhou 310003, China.

^ǂ^Haifeng Lu and Zhigang Ren contributed equally to this work.

^⃰^ Corresponding authors: Lanjuan Li, State Key Laboratory Diagnosis and Treatment for Infectious Disease, Collaborative Innovation Center for Diagnosis and Treatment of Infectious Diseases, The First Affiliated Hospital, School of Medicine, Zhejiang University, 79 Qingchun Road, Hangzhou 310003, P.R. China. Phone: +86571-87236456. Fax: +86571-87236456. E-mail: [ljli@zju.edu.cn](mailto:ljli@zju.edu.cn).

**Supplementary information**

**Supplementary table S1**. Questionnaire: Data for subjects participating in the investigation of the tongue coat microbiome

**Translated from Chinese**

**Data for subjects participating in the investigation of the tongue coat microbiome**

**Name：	sex：**F/M	**Date of birth (**month-year**)：**

**Telephone number(s)：	Sample NO:**

| Data of follow-up(Y-M-D) | | |  | | |
| --- | --- | --- | --- | --- | --- |
| Way of follow- up | | | 1. outpatient 2 inpatient | | |
| A survey   of disease symptom | 1. [pancreas](http://www.youdao.com/w/pancreas/#keyfrom=E2Ctranslation), Liver , lung or previous related surgery  2. Obstruction of common bile duct  3.Basic diseases such as coronary disease, hypertension  4.Diabetes  5.Gastrointestinal bleeding  6.Co-infection with virus and bacterial infection  7.Symptoms of respiratory: shortness of breath, sputum production,wheezing or chest pain or discomfort.  8.Others | |  | | |
| X-ray examination | | | Examation day： | | |
|  |  |  |  | | |
| CT examination | | | Examation day： | | |
|  |  |  |  | | |
| Endoscopic ultrasonography and histopathologic examination | | | Examation day： | | |
|  |  |  |  | | |
| Oral Health Screening | | | Examation day： | | |
|  |  |  | 1. [periodontitis](http://dict.youdao.com/w/periodontitis/) □  2. oral ulcer □  3. Cavities □  4. Tongue coat:  5. Canker sores  6. Others: ______ | | |
| Biochemical blood routine inspection | | | Examation day： | | |
|  |  |  |  | | |
| Physical sign | Blood pressure（mmHg） | |  | | |
|  | Weight（kg） | |  | | |
|  | [Height](javascript:void(0);) (cm) | |  | | |
|  | Body mass index （BMI） | |  | | |
|  | Others | |  | | |
| Lifestyles | Daily consumption of cigarettes | |  | | |
|  | Daily consumption of  alcohol | |  | | |
|  | Others (including brush habits, sleep habits, food habits and other drugs) | |  | | |
|  | Fitness habits | | Form: Time: | | |
|  | Dietaryhabits | Yoghurt |  | | |
|  |  | staple |  | | |
|  | Psychological quality | | 1.better 2.good 3.worse □ | | |
|  | Treatment compliance | | 1.better 2.good 3.worse □ | | |
| Drugs | Antibiotics | |  | | |
|  | Probiotics | |  | | |
|  | Or others | |  | | |
|  | Drugs 1: (name) | |  | | |
|  | Drug dosage | | Times/Day: Dosage/time: mg | | |
|  | Drugs 2:(name) | |  |  |  |
|  | Drug dosage | | Times/Day: Dosage/time: mg | | |
|  | Drugs 3:(name) | |  | | |
|  | Drug dosage | | Times/Day: Dosage/time: mg | | |
|  | Others | |  | | |
| Other need to supplement | | |  | | |
| Patients signature | | |  | | |
| Doctor signature | | |  | | |

**Supplementary Figures S1-S3**.

**Fig. S1** Principal coordinates analysis (PCoA) using unweighted UniFrac scores for the microbiota of PCT (red) and HCT (green) samples using different methods for calculating distances (A–C) Hellinger distance, (D–F) Jensen–Shannon divergence analysis and (G–I) the Spearman coefficient distance. Each symbol represents a sample. The variance explained by the PCoA is indicated in parentheses on the axes (circles highlight the clustering of the oropharyngeal microbiomes of the PC group).

**Fig. S2** Comparison of phyla of the microbiomes between PCT and HT. (a) Comparison of the abundances of bacterial phyla of each sample; (b) Comparison of the average abundance of each bacterial phylum in the PCT and HT, respectively; (c) Significant differences among the abundances of discriminatory phyla between PCT (red) and HT (blue); Box parameters, the “+ ” symbol represents median value, and the upper and lower ranges of the box represent the 75% and 25% quartiles, respectively. *p* values were calculated using the nonparametric Mann–Whitney test and were shown in Supplementary Datasets S2_a. Significant differences by *p < 0.05; **p < 0.01 and ***p < 0.001. PCT, pancreatic cancer patients tongue coat; HT, healthy subjects tongue coat.

**Fig. S3** Principal coordinates analysis (PCoA) using unweighted UniFrac scores for the microbiota of PCT (red) and LCT (blue) samples using different methods for calculating distances (A–C) Hellinger distance, (D–F) Jensen–Shannon divergence analysis and (G–I) the Spearman coefficient distance. Each symbol represents a sample. The variance explained by the PCoA is indicated in parentheses on the axes (circles highlight the clustering of the oropharyngeal microbiomes of the PC group).

**Supplementary Datasets S1.** Sequences determined using the MiSeq System (Illumina). Supplementary Datasets S1_a, Annotation of all OTU detected using 16-s rDNA gene sequencing; Supplementary Datasets S1_b, The relative abundance of 103 bacterial genera in each microbiome which retained after 2100 reads subsumpled randomly; Supplementary Datasets S1_c, Species-level OTUs and species richness and diversity estimates of each microbiome.

**Supplementary Datasets S2.** Statistical analysis of tongue coat microbiomes using the nonparametric Mann–Whitney test for phyla between PC patients and healthy controls (Supplementary Datasets S2_a), family between PC patients and healthy controls (Supplementary Datasets S2_b), genera between PC patients and healthy controls (Supplementary Datasets S2_c), genera between PC patients and liver cancer patients(Supplementary Datasets S2_d), classes between PC patients and healthy controls (Supplementary Datasets S2_e), orders between PC patients and healthy controls (Supplementary Datasets S2_f).

**The detailed script of microbial marker identification**

if(@ARGV<5){

print "RandomForeat2ROC.pl otu_table feature_importance_file Wilcox_file group top_number\n";

print "The otu_table should be the subset table, contain two group samples\n";

exit;

}

my $otu_table = shift;

my $feature_File = shift;

my $Wilcox = shift;

my $group = shift;

my $top_number = shift;

open(OTU,"<$otu_table") or die;

my $head = <OTU>;

chomp $head;

my %abundance;

my @headers = split /\t/,$head;

while(<OTU>){

chomp $_;

my @line = split /\t/,$_;

for(my $i=1;$i<@line;$i++){

${$abundance{$line[0]}}{$headers[$i]} = $line[$i];

}

}

close(OTU);

`sed 's/\"//g' $Wilcox >wilcox.tmp.xls`;

open(WILCOX,"<wilcox.tmp.xls") or die;

my $head_w = <WILCOX>;

chomp $head_w;

my @headers_w = split /\t/,$head_w;

$headers_w[1] =~ /**\(**(**\S**+)**\)**/;

$headers_w[1] = $1;

#print "$headers_w[1]\n";

$headers_w[3] =~ /**\(**(**\S**+)**\)**/;

$headers_w[3] =	$1;

my @G1; my @G2;

my %abundance_G1; my %abundance_G2;

while(<WILCOX>){

chomp $_;

my @line = split /\t/,$_;

$abundance_G1{$line[0]} = $line[1];

$abundance_G2{$line[0]} = $line[3];

#print "$line[0]\tccc\t$line[1]\t$line[3]\t$_\n";

if($line[1] > $line[3]){

#print "$line[0]\t$line[1]\t$line[3]\n";

if(@G1 <= $top_number){

$G1[@G1] = $line[0];

}

}else{

if(@G2 <= $top_number){

$G2[@G2] = $line[0];

}

}

}

close(WILCOX);

my %groups;

open(GROUP,"<$group") or die;

while(<GROUP>){

chomp $_;

my @line = split /\t/,$_;

$groups{$line[0]} = $line[1];

}

close(GROUP);

my @RF1; my @RF2;

my $mark_index=0;

open(RF,"<$feature_File") or die;

<RF>;

while(<RF>){

chomp $_;

$mark_index++;

my @line = split /\t/,$_;

if($mark_index<= $top_number){

if($abundance_G1{$line[0]} >$abundance_G2{$line[0]}){

#if(@RF1 < $top_number){

$RF1[@RF1] = $line[0];

#}

}else{

#if(@RF2 < $top_number){

$RF2[@RF2] = $line[0];

#}

}

}

}

close(RF);

my $aaa = join("\t",@RF1);

my $bbb = join("\t",@RF2);

open(COMB,">combination.details.xls") or die;

open(ROC,">ROC.combination.xls") or die;

print COMB "$headers_w[1] Group top $top_number OTUs: $aaa\n";

print COMB "$headers_w[3] Group top $top_number OTUs: $bbb\n";

print COMB "Combination\t$headers_w[1] richness\t$headers_w[3] richness\n";

my $tmp = join("\t",@headers);

print ROC "$tmp\n";

print ROC "GROUP\t";

my @Mark;

for($i=1;$i<@headers;$i++){

$mark[@mark] = $groups{$headers[$i]};

}

my $tmp2 = join("\t",@mark);

print ROC "$tmp2\n";

my @details_RF1; my @details_RF2;

for($m=0;$m<=@RF1;$m++){

for($n=0;$n<=@RF2;$n++){

if($m+$n>0){

my $tmp3 = "MIA_".$headers_w[1].$m.$headers_w[3].$n;

print ROC "$tmp3\t";

print COMB "$tmp3:\t";

my @combb1;

my @abund_tmp1; my @abund_comb1;

if($m>0){

for($p=0;$p<=$m-1;$p++){

$combb1[@combb1] = $RF1[$p];

for($i=1;$i<@headers;$i++){

$abund_tmp1[$i-1] += ${$abundance{$RF1[$p]}}{$headers[$i]};

#$combb1[@combb1] = $RF1[$p];

}

}

$p--;

for($i=1;$i<@headers;$i++){

$abund_comb1[$i-1] = $abund_tmp1[$i-1]/($m-$p);

}

}else{

for($i=1;$i<@headers;$i++){

$abund_comb1[$i-1] = 0;

$combb1[0] ="None";

}

}

my @abund_tmp2; my @abund_comb2;

my @combb2;

if($n>0){

for($q=0;$q<=$n-1;$q++){

$combb2[@combb2] = $RF2[$q];

for($i=1;$i<@headers;$i++){

$abund_tmp2[$i-1] += ${$abundance{$RF2[$q]}}{$headers[$i]};

#$combb2[@combb2] = $RF2[$q];

}

}

$q--;

for($i=1;$i<@headers;$i++){

$abund_comb2[$i-1] = $abund_tmp2[$i-1]/($n-$q);

}

}else{

for($i=1;$i<@headers;$i++){

$abund_comb2[$i-1] = 0;

$combb2[0] ="None";

}

}

my @abund_MIA;

for($i=1;$i<@headers;$i++){

$abund_MIA[$i-1] = $abund_comb1[$i-1] - $abund_comb2[$i-1];

}

my $tmp_RF = join("\t",@abund_MIA);

print ROC "$tmp_RF\n";

my $tmp_comb1 = join(";",@combb1);

my $tmp_comb2 = join(";",@combb2);

print COMB "$tmp_comb1\t$tmp_comb2\n";

}

}

}

`more ROC.combination.xls |awk -F"\t" '{for(i=1;i<=NF;i++){a[FNR,i]=\$i}}END{for(i=1;i<=NF;i++){for(j=1;j<=FNR;j++){printf a[j,i]"\t"}print ""}}' >ROC.combination.T.xls`;

open(RCMD,">cmd.r") or die;

print RCMD "

library(pROC)

data <-read.table(file=\"ROC.combination.T.xls\",header=T,check.names=FALSE,sep=\"\\t\")

comb <-dim(data)[2]-1

a<-c(rep(\"NA\",comb-2))

b<-c(rep(0,comb-2))

for(i in 3:comb){

name <- colnames(data)[i]

pdf(paste(name,\".pdf\",sep=\"\"))

roc <- roc(data[,2],data[,i],plot=T,col=\"red\",print.thres=T,print.auc=T)

dev.off()

a[i-2] <- colnames(data)[i]

b[i-2] <- roc\$auc

c<- data.frame(a,b)

}

colnames(c) <- c(\"Combation\",\"ROC_AUC\")

write.table(c,\"ROC.combination.AUC.xls\",sep=\"\t\")

";

`R --restore --no-save < cmd.r`;
